# Cell Line Models for Acquired Resistance to First-Line Osimertinib in Lung Cancers—Applications and Limitations

**DOI:** 10.3390/cells10020354

**Published:** 2021-02-09

**Authors:** Shuta Ohara, Kenichi Suda, Tetsuya Mitsudomi

**Affiliations:** Division of Thoracic Surgery, Department of Surgery, Kindai University Faculty of Medicine, Osaka-Sayama 589-8511, Japan; 154148@med.kindai.ac.jp (S.O.); mitsudom@med.kindai.ac.jp (T.M.)

**Keywords:** osimertinib, acquired resistance, cell line models, *EGFR* mutation, non-small-cell lung cancer, bypass pathway, epithelial to mesenchymal transition (EMT)

## Abstract

Epidermal growth factor receptor (EGFR) tyrosine kinase inhibitors (TKIs) are first-line drugs for lung cancers with activating *EGFR* mutations. Although first- and second-generation EGFR-TKIs were standard first-line treatments, acquired resistance (AR) to these drugs is almost inevitable. Cell line models have been widely used to explore the molecular mechanisms of AR to first- and second-generation EGFR-TKIs. Many research groups, including ours, have established AR cell lines that harbor the *EGFR* T790M secondary mutation, *MET* gene amplification, or epithelial–mesenchymal transition (EMT) features, which are all found in clinical specimens obtained from TKI-refractory lesions. Currently, many oncologists prescribe osimertinib, a third-generation EGFR-TKI that can overcome T790M-mediated resistance, as a first-line TKI. Although few clinical data are available about AR mechanisms that arise when osimertinib is used as a first-line therapy, many research groups have established cell lines with AR to osimertinib and have reported on their AR mechanisms. In this review, we summarize the findings on AR mechanisms against first-line osimertinib obtained from analyses of cell line models.

## 1. Introduction

Lung cancer is the leading cause of cancer-related deaths worldwide. Intensive molecular analyses of lung cancers, especially in lung adenocarcinomas, have identified several mutually exclusive aberrations that occur in proto-oncogenes. Subsequently, clinical trials have shown that orally available kinase inhibitors are highly active against tumors that harbor these driver gene mutations. Therefore, based on the results of many clinical trials, current guidelines recommend the first-line use of kinase inhibitors when treating unresectable lung cancers with mutations in epidermal growth factor receptor (*EGFR*), anaplastic lymphoma kinase (*ALK*), *ROS1*, *BRAF*, neurotrophic receptor tyrosine kinase 1/2/3 (*NTRK1/2/3*), or *MET* [1,2,3,4,5,6].

*EGFR* mutations are the most common molecular aberration found in lung cancers. They are found in ~50% of lung cancers in East Asians and ~15% of those in Caucasians [7]. The standard of care for lung cancers with *EGFR* mutations is EGFR tyrosine kinase inhibitor (TKI) monotherapies. The so-called first-generation EGFR-TKIs (gefitinib and erlotinib) and a second-generation EGFR-TKI (afatinib) were used as first-line EGFR-TKIs until the approval of a third-generation EGFR-TKI (osimertinib) for first-line use.

Despite the dramatic responses of lung cancers with *EGFR* mutations to EGFR-TKIs, the emergence of acquired resistance (AR) is almost inevitable [8]. To understand and to overcome AR to EGFR-TKIs, researchers have explored resistance mechanisms by analyzing tumor specimens obtained from patients who developed AR to an EGFR-TKI [9,10,11] or by analyzing cell lines that acquired resistance to an EGFR-TKI in vitro by chronic exposure to the drug [10,11,12]. Both of these research paths have identified many AR mechanisms to first- and second-generation EGFR-TKIs, including T790M *EGFR* secondary mutation, as the most common mechanism, followed by *MET* gene amplification, *ERBB2* gene amplification, small-cell lung cancer (SCLC) transformation, and the acquisition of epithelial to mesenchymal transition (EMT) features [13].

Osimertinib is a third-generation EGFR-TKI that was designed to overcome T790M-mediated resistance while sparing wild-type EGFR [14]. Osimertinib was originally approved as a second-line EGFR-TKI for patients with lung cancer with *EGFR* mutations that acquired resistance to first or second-generation EGFR-TKIs through the T790M secondary mutation. However, the FLAURA trial showed that first-line osimertinib is superior to first-generation EGFR-TKIs in both progression-free and overall survivals [15]. Currently, many oncologists use osimertinib as the first-line EGFR-TKI.

However, developing resistance to first-line osimertinib is also inevitable. In this review, we summarize published findings regarding AR mechanisms to first-line osimertinib, with a particular focus on resistance mechanisms that were identified using cell line models.

## 2. Identifying Mechanisms of Resistance to EGFR-TKIs through Cell Line Models

Cell line models are widely used in lung cancer research to evaluate drug resistance mechanisms [16,17,18,19,20,21]. Engelman et al. were the first who successfully identified a novel AR mechanism against EGFR-TKI—*MET* gene amplification—by using a cell line model in 2007. They established a gefitinib-resistant cell line model with *MET* gene amplification by exposing a gefitinib-sensitive lung cancer cell line (HCC827) with an activating *EGFR* mutation (exon 19 E746_A750 del) to increasing concentrations of gefitinib. More importantly, they also used clinical specimens to show that *MET* gene amplification was detected in 4 of 18 (22%) of the lung cancer specimens that had developed AR to gefitinib or erlotinib [10]. The authors also showed that EGFR-TKI resistance mediated by *MET* gene amplification can be overcome by the combination of a MET-TKI plus gefitinib.

The *EGFR* T790M secondary mutation is the most common mechanism of AR to first- or second-generation EGFR-TKIs found in clinical specimens [22]. PC9 cells (exon 19 E746_A750 del) are a lung cancer cell line that often acquires resistance to EGFR-TKIs through T790M secondary mutation [22]. Hata et al. reported that PC9 cells contain a pre-existing minor subclone with the T790M mutation. The T790M secondary mutation was also induced in single-cell PC9 clones that were established to eliminate the pre-existing minor clone with a T790M mutation [23]. Furthermore, T790M-mediated AR to first-generation EGFR-TKIs has been reported in other cell lines, such as HCC4006 (exon 19 del L747_A750 ins P) [24] and HCC827 cells [11], which reflects the very high frequency of this resistance mechanism.

As another AR mechanism against first-generation EGFR-TKIs, in 2011, we established and reported an AR cell line model derived from HCC4006 cells with EMT features [12]. EMT has also been reported as an AR mechanism against EGFR-TKIs in clinical specimens [25,26]. Although *ERBB2* gene amplification and SCLC transformation have been reported as AR mechanisms against EGFR-TKIs in clinical settings, as far as we know, no cell line model has been developed with either *ERBB2* gene amplification or SCLC transformation by using chronic exposure to EGFR-TKIs. Many groups have reported potential AR mechanisms against first- or second-generation EGFR-TKIs using cell line models, such as activating β-catenin [27] or the insulin-like growth factor receptor (IGF-1R) [28,29,30,31]. However, the roles of these molecules in AR against EGFR-TKIs in clinical settings are unclear.

## 3. Cell Line Models Used to Analyze Resistance Mechanisms to First-Line Osimertinib

### 3.1. Search Criteria for Published Studies

To identify published articles that analyzed AR mechanisms against first-line osimertinib using cell line models, we systematically searched PubMed for relevant studies as of December 1, 2020. Our search criteria included the following terms: “osimertinib,” “resistance,” “lung cancer,” and “cell line” or “cell lines.” We also manually scanned the reference lists of selected articles for additional eligible publications. We finally identified 29 relevant papers [32,33,34,35,36,37,38,39,40,41,42,43,44,45,46,47,48,49,50,51,52,53,54,55,56,57,58,59,60]. We excluded 2 of these papers [47,49], as they used osimertinib-resistant cell lines that had been established in other studies included in our survey.

**Figure 1:**
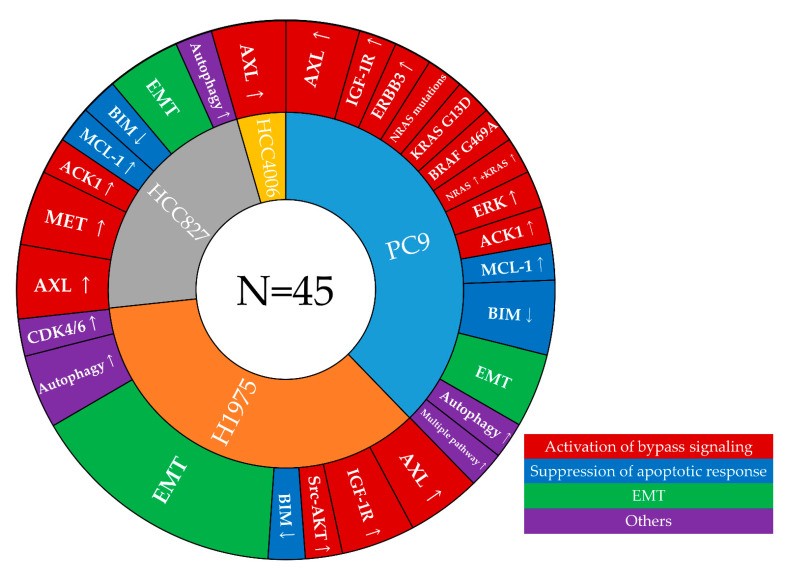
Mechanisms of acquired resistance to first-line osimertinib that were identified in cell line models [32,33,34,35,36,37,38,39,40,41,42,43,44,45,46,48,50,51,52,53,54,55,56,57,58,59,60]. This graph is color-coded by cell line models and mechanisms of resistance to first-line osimertinib. EGFR, epidermal growth factor receptor; AXL, AXL receptor tyrosine kinase; ERBB3, human epidermal growth factor receptor-3; IGF1R, insulin-like growth factor-1 receptor; TGF, transforming growth factor; ACK1, activated Cdc42-associated kinase-1; CDK4/6, cyclin-dependent kinase; BCL-2, B-cell lymphoma-2; YAP, yes-associated protein; ANKRD1, ankyrin repeat domain-1; IRE1α, inositol-requiring enzyme-1α.

In the 27 remaining studies, 17 AR cell lines were established from PC9 cells, 16 from H1975 cells (L858R plus T790M, a cell line model with primary resistance to first- or second-generation EGFR-TKIs), 10 from HCC827 cells, and 2 from HCC4006 cells (Figure 1). Twenty-five studies used chronic exposure to osimertinib at increasing concentrations, one study [57] used chronic exposure to osimertinib at a consistent concentration, and one study [52] used two different exposures (increasing concentration and a consistent concentration). We classified the AR mechanisms into five categories: (a) aberration of EGFR itself, (b) activation of bypass signaling, (c) suppression of apoptosis, (d) EMT, and (e) other mechanisms.

### 3.2. Mechanisms of Resistance to First-Line Osimertinib That Were Identified in Cell Line Models

#### 3.2.1. Aberration of EGFR Itself—On-Target Resistance Mechanism

As described above, the *EGFR* T790M secondary mutation is the most common AR mechanism against first- and second-generation EGFR-TKIs, and many TKI-resistant cell line models acquired this secondary mutation. Some *EGFR* secondary mutations (C797X: 7%, L718Q + C797S: 1%, L718Q + ex20ins: 1%, S768I: 1%) have been reported as AR mechanisms against first-line osimertinib in clinical settings [61]. However, we could not find a cell line model with AR against first-line osimertinib based on an *EGFR* secondary mutation.

#### 3.2.2. Activation of Bypass Signaling

**Figure 2:**
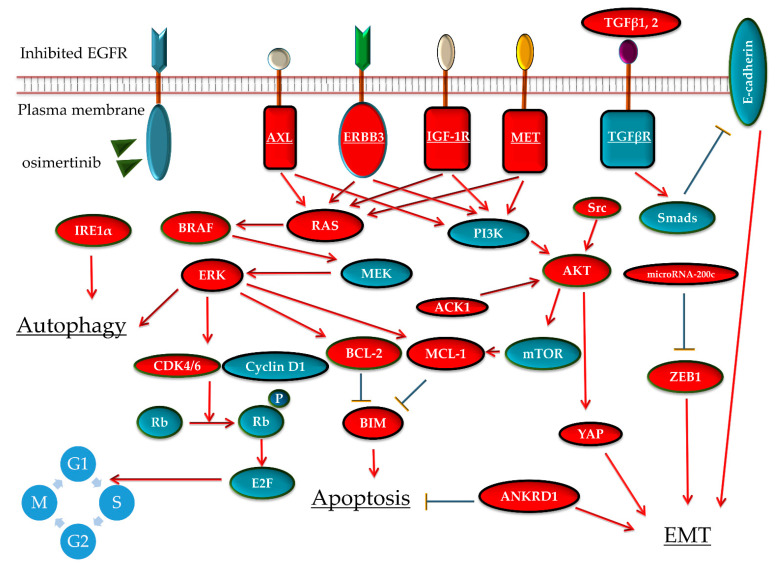
Scheme of the signal pathway and molecules that reportedly cause osimertinib resistance—AXL [39,41,52], HER3 [45], IGF1R [51,57], MET [33,52], TGFβ1 [40], TGFβ2 [59], BRAF [43], RAS [32,35], ERK [37], Src-AKT [35], ACK1 [48], CDK4/6 [55], BCL-2 [56], MCL-1 [34], BIM [34,50], YAP [54], ANKRD1 [36], microRNA-200c [46], ZEB1 [46,58], and IRE1α [38] (highlighted in red)—in cell line models treated with osimertinib. EGFR, epidermal growth factor receptor; AXL, AXL receptor tyrosine kinase; ERBB3, human epidermal growth factor receptor-3; IGF1R, insulin-like growth factor-1 receptor; MET, MET proto-oncogene, receptor tyrosine kinase; TGF, transforming growth factor; ACK1, activated Cdc42-associated kinase-1; CDK4/6, cyclin-dependent kinase; BCL-2, B-cell lymphoma-2; YAP, yes-associated protein; ANKRD1, ankyrin repeat domain-1; IRE1α, inositol-requiring enzyme-1α.

The activation of other receptor tyrosine kinases is a common AR mechanism against EGFR-TKIs. Reportedly, in vitro analyses have implicated several receptor tyrosine kinases in AR mechanisms against first-line osimertinib. These receptor tyrosine kinases are able to activate downstream pathways related to cell proliferation and survival, instead of the inhibited EGFR. Molecules and pathways that may have roles in AR to first-line osimertinib are summarized in Figure 2.

##### *MET* Gene Amplification

Similar to the AR mechanisms against first- or second-generation EGFR-TKIs [10,11], *MET* gene amplification is reported to be a resistance mechanism against first-line osimertinib in studies that used HCC827 cells [33]. This is reasonable because HCC827 cells have a pre-existing minor clone with *MET* gene amplification [62], and this cell line has repeatedly acquired resistance to several EGFR-TKIs through *MET* gene amplification [10,11,22].

In analyses of clinical specimens, *MET* gene amplification is a common AR mechanism against first-line osimertinib. For example, AR mechanisms were explored using circulating tumor DNA analysis in patients who were enrolled in the phase-III FLAURA study, which compared the efficacy of first-line osimertinib to first-generation EGFR-TKIs (gefitinib or erlotinib). In this study, *MET* gene amplification was reportedly the most common AR mechanism (*n* = 14/91; 15%) in the osimertinib arm [61]. Another study reported *MET* gene amplification as the AR mechanism against first-line osimertinib in 66% (*n* = 6/9) of their patients [63]. Currently, several clinical trials that co-target MET are ongoing for *EGFR* mutated non-small cell lung cancer (NSCLC) patients who acquired resistance to osimertinib; these include savolitinib plus osimertinib (SAVANNAH) or tepotinib plus osimertinib (INSIGHT 2).

##### AXL Activation

Increased AXL expression was reported by independent research groups as a resistance mechanism against first-line osimertinib using cell line models. AXL is a receptor tyrosine kinase that binds to a ligand (Gas6) and potentiates survival signaling by activating the PI3K–Akt and RAS–RAF–MEK–ERK pathways [64]. Originally, AR mediated by AXL was reported in HCC827 cells that acquired resistance to the first-generation EGFR-TKI, erlotinib [65]. As a resistance mechanism against first-line osimertinib, cells that acquired resistance through the activation of AXL and MET were established from HCC827 cells; a combination of an AXL/MET dual inhibitor (CB469) plus osimertinib could overcome the resistance [52]. Additionally, another group reported establishing acquired resistant PC9, H1975, and HCC4006 cell lines with AXL activation. The authors also explored the mechanism of AXL activation in osimertinib-resistant cells; mechanically, STC2 enhanced the AXL promoter activity by increasing the phosphorylation of c-Jun, which is an indispensable transcription factor that transactivates AXL [41]. Osimertinib-resistant cells with AXL activation were also established from PC9, HCC827, HCC4006, and H1975 cells; these resistant cells were sensitive to the combination of osimertinib plus cabozantinib [39].

Although AXL-mediated resistant cell lines have been repeatedly established in vitro, to our knowledge, there are no reported clinical cases in which AXL activation developed after first-line osimertinib treatment failure. However, several studies have reported high AXL expression in tumor specimens obtained after first or second EGFR-TKI treatment failure [65,66,67]. Another study reported that the response rate for osimertinib is relatively low (66.7%) in patients whose lung cancer has an EGFR mutation and a high AXL expression (IHC score 3+) [68]. These results, together with repeated development of osimertinib-resistant, AXL-activated cell lines, imply that AXL activation could be an AR mechanism against first-line osimertinib. However, this should be confirmed clinically. In addition to the AXL inhibitors described above, many AXL inhibitors (e.g., BGB324, TP-0903, ONO-7475, or DS-1205b) have been investigated in preclinical studies; therefore, we anticipate the clinical application of these agents in the near future.

##### ERBB3 Activation

ERBB3 is one of four members of the human ERBB family. ERBB3 lacks intrinsic tyrosine kinase activity but contains six YXXM-consensus binding sites for the SH2 domains of the p85 regulatory subunit of PI3K [69]. It therefore potentiates survival by activating PI3K/Akt signaling [70]. Yonesaka et al. reported the upregulation of ERBB3 in PC9 cells as a mechanism of AR to osimertinib. The authors also reported that an anti-HER3 antibody drug (U3-1402) might serve as a novel therapy that can overcome AR mediated by ERBB3 activation [45]. A clinical trial of U3-1402 plus osimertinib for *EGFR* mutated NSCLC patients who acquired resistance to osimertinib has just been posted (NCT04676477). So far, we have found no clinical cases with ERBB3 activation after first-line osimertinib treatment failure.

##### IGF-1R Activation

IGF-1R also potentiates survival by activating PI3K–Akt signaling and RAS–RAF–MEK–ERK signaling [71]. The role of IGF-1R as an AR mechanism against first- or second-generation EGFR-TKIs has been reported previously [29,30,31]. Two independent research groups, using H1975 cells, both reported that IGF-1R activation is an AR mechanism against osimertinib, and that combining osimertinib with an IGF-1R inhibitor (linsitinib) can overcome AR to osimertinib through IGF-1R activation [51,57]. Among clinical cases, Manabe et al. reported that 4 of 6 (66.7%) patients eventually showed elevated IGF2 after first-line osimertinib treatment failure [51].

##### ERK or AKT Pathway Reactivation

The activation of signaling downstream of EGFR, including the PI3K–AKT, RAS–RAF–MEK–ERK, and JAK–STAT pathways, is a common AR mechanism against EGFR-TKIs. For example, we and other groups have reported that PTEN downregulation can activate the PI3K–AKT pathway as a resistance mechanism against first-generation EGFR-TKIs [72,73,74]. As AR mechanisms against first-line osimertinib, Src-mediated activation of AKT is reported as a mechanism of resistance in H1975 cells [35]. Another group reported that increased ACK1, which phosphorylates AKT and activates the AKT pathway [75], is an AR mechanism against osimertinib in PC9 and HCC827 cells, and that osimertinib plus an ACK1 inhibitor ([R]-9b) might overcome osimertinib resistance [48]. In addition, many groups have suggested that the RAS–RAF–MEK–ERK pathway is involved in the acquisition of resistance to first-line osimertinib in vitro.

Monica et al. established a PC9 cell line with AR to osimertinib and found that the resistant cells had acquired a *BRAF* G469A mutation. The authors also found that *BRAF* G469A, which is a class II mutation, maintained the constitutive activation of the ERK pathway and that combining selumetinib or trametinib with osimertinib can overcome resistance from the *BRAF* G469A mutation [43].

The *BRAF* mutation is a rare driver mutation in lung adenocarcinoma (1~2%) that is mutually exclusive with *EGFR* mutations [1,76,77,78]. V600E is the most common *BRAF* mutation. It is present in about half of NSCLCs with *BRAF*-activating mutations. The acquisition of a *BRAF* V600E mutation has been reported after the failure of first-line osimertinib treatment [79,80,81,82]. Additionally, BRAF activation through *BRAF* gene fusion has also been reported as a resistance mechanism against osimertinib in clinical specimens [83].

The activation of RAS family members has also been reported in cell line models as an AR mechanism against first-line osimertinib. Nukaga et al. reported that PC9 cells that acquired resistance to osimertinib developed a *KRAS* G13D mutation [35]. Another group reported that the upregulation of KRAS expression was a mechanism of AR to osimertinib in PC9 cells [32]. In a study in which osimertinib-resistant cells with ERK reactivation were established from PC9 cells, no specific mechanism of ERK reactivation was detected, but the *HRAS* G13R mutation partially contributed to ERK reactivation in these cells [37]. Aberration in another RAS gene, NRAS, may also contribute to AR to first-line osimertinib. Eberlein et al. reported that upregulation of *NRAS* RNA expression, and *NRAS* mutations (*NRAS* E63K, *NRAS* G12V, *NRAS* G12R) are mechanisms of AR to osimertinib in PC9 cells, and that the combination of osimertinib plus an MEK inhibitor (selumetinib) can overcome the osimertinib resistance [32].

Many groups have reported finding *KRAS* mutations (G12C, G12D, G12S), *BRAF* mutations (D594N, V600E), *BRAF* fusions, *PIK3CA* mutations (E545K, E542K, R88Q, N345K, E418K), and PTEN loss in specimens from patients who developed AR against second-line osimertinib [61,84,85,86,87,88,89,90,91]. Furthermore, a recent study reported that *PIK3CA* mutations (E453K, E545K, H1047R; 7%), *BRAF* V600E mutations (3%), and *KRAS* mutations (G12D/C, A146T; 3%) were detected in specimens from patients who developed AR to first-line osimertinib [61]. Therefore, ERK reactivation through several mechanisms may be an important and common AR mechanism against both first- and second-line osimertinib therapy.

#### 3.2.3. Suppression of Apoptotic Response

Apoptotic response is the final step when *EGFR*-mutated lung cancer cells are killed by an EGFR-TKI. Therefore, dysregulation of apoptosis might be reasonably supposed as a cause of inherent or acquired resistance to EGFR-TKIs. For example, the polymorphism of BCL-2-like 11 (*BIM*), a pro-apoptotic member of the BCL2 family, is reportedly sufficient to confer intrinsic resistance to first-generation EGFR-TKIs in lung cancers [92]. Although we could not find a paper that described the role of *BIM* polymorphism in first-line osimertinib efficacy, a recent study suggested that *BIM* polymorphism is correlated with worse outcomes in patients who received osimertinib after first- or second-generation EGFR-TKI failure due to a T790M secondary mutation [93].

As an AR mechanism against first-line osimertinib in vitro, the upregulation of MCL-1 (another anti-apoptotic protein) and downregulation of BIM was reported as the mechanism of AR to osimertinib in experiments that used PC9 and HCC827 cells. The authors showed that MEK inhibitors (GSK1120212 or PD-0325901) combined with osimertinib reduced MCL-1 expression and could overcome the resistance [34]. The same group reported in a later study that honokiol (a natural product isolated from the bark, seed cones, and leaves of trees belonging to the genus *Magnolia*) plus osimertinib enhanced MCL-1 reduction by suppressing ERK-dependent MCL-1 phosphorylation [47], and that the histone deacetylase inhibitor (LBH589) enhanced the effects of osimertinib in the resistant cells by decreasing levels of p-ERK and increasing BIM levels [49]. Another group reported that in experiments using PC9 and H1975 cells, BIM downregulation was the mechanism of AR to osimertinib. The authors showed that combining aspirin with osimertinib could overcome this resistance by promoting BIM-dependent apoptosis [50]. In addition, significant upregulation of the BCL-2 protein was reported in osimertinib-resistant H1975 cells, compared to their H1975 parental cells. The authors of this study showed that adding a BCL-2 inhibitor (ABT263 or ABT199) to osimertinib could overcome the resistance mediated by BCL-2 overexpression [56].

#### 3.2.4. EMT

EMT is a process by which epithelial cells lose their cell polarity and cell–cell adhesion and acquire mesenchymal phenotypes that are associated with increased migratory and invasive properties, as well as drug resistance. We were one of the first groups to report the involvement of EMT in the acquisition of resistance to EGFR-TKIs in 2011 [12]. Several later studies indicated that the HCC4006 and H1975 cell lines often acquire resistance to EGFR-TKIs through EMT [94]. The overexpression of the ankyrin repeat domain-1 (ANKRD1), which is associated with the EMT process and anti-apoptosis, was identified in afatinib- and osimertinib-resistant cells established from PC9 and HCC827 cells. The authors also observed that imatinib could inhibit ANKRD1 expression, resulting in the restoration of sensitivity to afatinib and osimertinib in EGFR-TKI-resistant cells [36]. Another group established osimertinib-resistant cell lines from PC9 and HCC827 cells and reported that increased TGFβ1 induces high integrin β3 expression, which in turn induces the cells to revert to a mesenchymal phenotype. The authors also reported that combining osimertinib with a TGFβR1 inhibitor (SB-431542) could overcome the osimertinib resistance mediated through this pathway [40]. Other studies that reported the involvement of EMT in acquiring resistance to first-line osimertinib used H1975 cells [13,46,53,54,58,59,60], which were destined to develop EMT as an acquired resistance mechanism as described in our previous study [94]. Among mechanisms of EMT induction, increased ZEB1 expression [58], or decreased microRNA-200c in combination with increased ZEB1 expression [46] have been reported. The latter study also reported that GSK-3 inhibitor (LY2090314) may circumvent EMT-associated resistance to osimertinib [46]. Other important inducers of EMT include TGFβ2 and NF-kB signaling. An NF-kB pathway inhibitor (BAY 11-7082) reportedly caused cell death in resistant cells with EMT features [59]. Other groups have reported the respective roles of the YAP–FOXM1 axis [54] and the Src pathway [13] as central regulators of EMT-associated osimertinib resistance.

Several studies have reported evidence of EMT in tumor specimens obtained from patients who developed AR to first-generation EGFR-TKIs [25,58]. We therefore expect further reports of EMT-mediated AR to first-line osimertinib in the near future.

#### 3.2.5. Other Mechanisms

Many studies have reported AR mechanisms against first-line osimertinib that cannot be classified into the above four categories. We summarize these mechanisms below.

Increased inositol-requiring enzyme-1α (IRE1α) expression was reported as an AR mechanism against osimertinib in HCC827 cell line model [38]. IRE1α affects endoplasmic reticulum (ER)-based stress signals [95,96]. As the ER regulates protein folding, under stress conditions, the accumulation of immature proteins provokes the ER stress response and boosts autophagy, resulting in autophagic cell death [95]. This process is mainly regulated via the IRE1α–JNK and PERK–eIF2α–ATF4 pathways [96]. The authors reported that osimertinib sensitivity was restored through the knockdown of IRE1α or treatment with an IRE1α inhibitor (STF-083010) [38]. The potential role of enhanced autophagy in AR against first-line osimertinib has been reported by other groups that used H1975 or PC9 models [42,53]. One study suggested that combining osimertinib with an autophagy inhibitor (CQ) could improve osimertinib cytotoxicity [42]. Li et al. reported that autophagy is a common mechanism in second-line osimertinib resistance in clinical specimens [42].

Codony-Servat et al. reported that the activation of multiple oncogenes (AKT, STAT3, YAP, AXL, IGF-1R, MET) and the upregulation of BCL-2 constitute a mechanism of AR to osimertinib in PC9 cells, and that the combination of osimertinib plus an Hsp90 inhibitor (luminespib) can overcome osimertinib resistance mediated by multiple pathways [44].

Increased CDK4 expression and phosphorylation of Rb (the down-stream molecule of CDK4/6) were reported in cells with AR to osimertinib established from H1975 cells. In this resistant model, the combination of CDK4/6 inhibitor (palbociclib) plus osimertinib overcame the resistance [55]. The CDK4- and CDK6-mono-phosphorylated Rb proteins and the phosphorylated Rb protein partially relieved Rb-mediated suppression of the family of E2F transcription factors [97]. These processes promote cell progression from the G_1_ to the S phase [97]. Notably, *CDK4/6* gene amplification was detected in 5% of clinical specimens obtained from patients who progressed after first-line osimertinib treatment [61].

### 3.3. Correlation of Resistance Mechanisms Identified in Cell-Line Models and Clinical Specimens

As described above, the *EGFR* T790M secondary mutation [13,26,98] is the most common AR mechanism against first- and second-generation EGFR-TKIs at ~57%. Many studies have reported cell lines that develop resistance through T790M mutations after chronic exposure to first- or second-generation EGFR-TKIs [23]. However, as far as we know, no cell line has been reported to have developed AR to first-line osimertinib through *EGFR* secondary mutations. This is consistent with the lower frequency (~10%) of *EGFR* secondary mutations in clinical specimens obtained after first-line osimertinib failure.

That being said, many studies have reported the activation of a bypass pathway in cell lines that acquired resistance to first-line osimertinib. Some of them, such as *MET* gene amplification and *KRAS/BRAF* mutations, have been identified in clinical specimens.

Analyses of clinical specimens have reported higher frequency of SCLC transformation as a resistance mechanism to osimertinib. Interestingly, however, no cell line model with AR has shown evidence of SCLC transformation, which suggests a limitation of cell line models.

## 4. Summary

In this review, we summarized numerous mechanisms of AR to first-line osimertinib exposure in cell line models. We described that some of the resistance mechanisms that were found in cell line models were also identified in clinical specimens obtained from patients who developed AR to EGFR-TKIs, including those who acquired resistance to first-line osimertinib. Lessons from the experiments of acquired resistance mechanisms to first- or second-generation EGFR-TKIs have suggested that cell line models are useful tools to find novel mechanisms of resistance. Therefore, we hope that the catalog of resistance mechanisms to first-line osimertinib identified in cell line models summarized in this review helps researchers to identify acquired resistance mechanisms to first-line osimertinib in clinical specimens in the near future.
